# Quantification of rhBMP2 in bioactive bone materials

**DOI:** 10.1093/rb/rbz038

**Published:** 2019-12-16

**Authors:** Huan Lian, Han Wang, Qianqian Han, Chunren Wang

**Affiliations:** Division of Biomaterials, Department of Medical Devices, National Institutes for Food and Drug Control, No. 31 Huatuo Road, Beijing 102629, China

**Keywords:** rhBMP-2 bioactivity quantification, statistical model fitting, R programming

## Abstract

Bone morphogenetic protein (BMP), belongs to transforming growth factor-β (TGF-β) superfamily except BMP-1. Implanting BMP into muscular tissues induces ectopic bone formation at the site of implantation, which provides opportunity for the treatment of bone defects. Recombinant human BMP-2 (rhBMP-2) has been used clinically, but the lack of standard methods for quantifying rhBMP-2 biological activity greatly hindered the progress of commercialization. In this article, we describe an *in vitro* rhBMP-2 quantification method, as well as the data analyzation pipeline through logistic regression in RStudio. Previous studies indicated that alkaline phosphatase (ALP) activity of C2C12 cells was significantly increased when exposed to rhBMP-2, and showed dose-dependent effects in a certain concentration range of rhBMP-2. Thus, we chose to quantify ALP activity as an indicator of rhBMP-2 bioactivity *in vitro*. A sigmoid relationship between the ALP activity and concentration of rhBMP-2 was discovered. However, there are tons of regression models for such a non-linear relationship. It has always been a major concern for researchers to choose a proper model that not only fit data accurately, but also have parameters representing practical meanings. Therefore, to fit our rhBMP-2 quantification data, we applied two logistic regression models, three-parameter log-logistic model and four-parameter log-logistic model. The four-parameter log-logistic model (adj-*R*^2^ > 0.98) fits better than three-parameter log-logistic model (adj-*R*^2^ > 0.75) for the sigmoid curves. Overall, our results indicate rhBMP-2 quantification *in vitro* can be accomplished by detecting ALP activity and fitting four-parameter log-logistic model. Furthermore, we also provide a highly adaptable R script for any additional logistic models.

## Introduction

Since Wozney cloned human bone morphogenetic protein (BMP) cDNA gene for the first time in 1988, nearly 20 species of human BMP have been discovered. BMPs belong to transforming growth factor-β (TGF-β) superfamily, which not only play a key role in bone development, regeneration and repair, but also induce differentiation of mesenchymal cells to form cartilage and bone [1]. Therefore, the study of BMP has important theoretical significance, as well as a broad prospect of clinical application. Human BMP-2 is a glycosylated, disulfide-bonded, dimeric protein with two major subunit species of 114 and 131 amino acids. rhBMP-2 binds to receptors on the surface of mesenchymal cells and causes cells to differentiate into cartilage and bone-forming cells [2]. There are attempts of constructing bioactive bone materials by applying rhBMP-2 to a matrix such as absorbable collagen sponge [3]. These biomaterials enjoy sound prospect, for example, surgical approach such as spinal fusion may be considered for treating severe disc degeneration [4]. Bone growth at the fusion site requires facilitation of bone grafts or substitutes harvested from the patients themselves (autograft, usually from the iliac crest), bone from another individual (allograft), or synthetic alternatives. However, harvesting autologous iliac crest bone graft can result in pain as well as complications such as infection, hematoma and meralgia paresthetica related to the donor site [5]. The synthetic alternatives became in great demand. In addition, the method of extracting BMP from bone tissue is inefficient and cumbersome. Consequently, gaining a wider access of BMP through genetic engineering has become an inevitable way for translational study. Different kinds of human BMP have been expressed in eukaryotic cells. Currently, there are few commercially available medicinal products licensed for use, such as dibotermin alfa (rhBMP-2) with considerably high price [6]. As a matter of fact, the progress of bioactive bone material commercialization has been slowed down due to the lack of standardized method to quantify rhBMP-2 in medical devices.

Without the regulation of standard quantification, the data interpretation for both *in vivo* and clinical rhBMP-2 researches can be confusing. For example, the early work in a rat bone-defect model shown that rhBMP-2 incorporated in a small-sized magnetic liposomes with an amount of ∼3 μg could effectively induce new bone formation [7]. Another work in a rabbit ulnar-defect model of rhBMP-2 with a novel carrier, poly (d, l-lac-tide-co-glycolide-)-coated gelatin sponge (PGS), indicated doses between 0.4 and 1 mg/cm^3^ could sufficiently treat all defects in a dose-dependent manner [8]. Besides, more studies were accomplished due to investigate suitable carrier and minimum effective dose on dogs and sheeps by applying doses ranging from 20 to 600 μg [9–11]. In clinical trials, it is reported that 1.5 mg/ml dose of rhBMP-2/ACS was utilized for the interbody fusion [12]. Another trial of porous biphasic calcium phosphate ceramic carrier suggested a solid fusion can be formed with minimum concentration of 1.4 mg/ml rhBMP-2 [13]. However, a mandibular replacement surgery applied a titanium mesh cage filled with bone mineral blocks and 7 mg rhBMP-2 also produced satisfied outcome [14]. Although such a huge difference of rhBMP-2 dosage was possibly resulted from multiple factors including but not limited to rhBMP-2 carriers, surgical subjects and implantation sites, it is suggested that rhBMP-2 protein level may not be an appropriate indicator when measuring rhBMP-2 functionality *in vivo* and clinically. Thus, we proposed that using the actual rhBMP-2 bioactivity instead of protein level may be a practical start to standardize the evaluation and application of rhBMP-2-loaded medical devices.

Previous studies suggested that rhBMP-2 dose-dependently induced alkaline phosphatase (ALP) activity in C2C12 cells and was able to specifically convert the differentiation pathway of the clonal myoblastic cell line into that of osteoblast lineage [15]. ALP is an enzyme found in several tissues throughout the body. The highest concentrations of ALP are present in the cells that comprise bone and the liver. In addition, there are several different forms of ALP-called isoenzymes, which depends on where in the body it is produced [16]. The ALP test has long been used in the diagnosis of bone problems such as rickets, osteomalacia and Paget’s disease [17]. Above all, ALP activity could serve as an indicator for quantifying rhBMP-2 bioactivity. p-Nitrophenyl Phosphate (PNPP) is a widely used substrate for detecting ALP in ELISA applications [18]. When ALP and PNPP are reacted, a yellow water-soluble reaction product is formed. This reaction product can sensitively and accurately absorb light at 405 nm. Thus, to initiate the standardization of measuring rhBMP-2 bioactivity in medical devices, an *in vitro* study of rhBMP-2 in C2C12 cell line was conducted.

For most *in vitro* active bioassays, non-linear reactions are often encountered. However, mass computational models exist for such data, which extremely adds to wet lab researchers’ bewilderment. The major concern for researchers has been chosen a potent statistic model that not only fit data accurately, but also have parameters representing practical meanings [19]. It is demonstrated that logistic models are applicable to a quantitative response showing a sigmoid log–dose relationship, especially the four-parameter log-logistic model [20]. Regression is a method for fitting a mathematical model, *y* = *f*(*x_k_*, *a_j_*) (*k* = 1, 2, …, *n*; *j* = 1, 2,…, *m*), when *y* consists of proportions, probabilities or binary coded data. We can decide whether there is a group of parameters, *a_j_* (*j* = 1, 2, … , *m*), can accurately describe the relationship between the dependent variable y and the independent variables *x_k_* (*k* = 1, 2, …, *n*) in the arithmetic equation. In particular, according to the least-square method, if any of the parameters *a_j_* met the condition,

$$\sum_{i=1}^{n}r_{i}^{2}=\sum_{i=1}^{n}{\left[f\left(\mathrm{xi};a1,a2,\ldots \mathrm{am}\right)-\mathrm{yi}\right]}^{2}=\min$$

then *y* = *f*(*x_k_*, *a_j_*) (*k* = 1, 2, …, *n*; *j* = 1, 2,…, *m*) is a statistically fitted regression model. The logistic regression fits a logistic curve,

$$\log\frac{y}{1-y}=w^{T}x+b$$

which is an S-shaped or sigmoid curve. The equation transformed due to the number of significant parameters. The random variable *X* is said to have a log-logistic distribution if *lnX* is logistic. The log-logistic distribution has long been used for modeling some survival data due to its non-monotonic property [21]. Here, we chose three-parameter log-logistic and four-parameter log-logistic models to fit our rhBMP-2 bioactivity data, because they were previously reported on immunoassay [22].

An R script has been written to facilitate the calculations, which include logistic curve-fitting as well as tests of goodness of fitting [23]. All parameters of both models were fitted by applying Gauss–Newton algorithm. The performance of fitted model was evaluated by *R*-squared (*R*^2^), adjusted *R*-squared (adj-*R*^2^) and residual standard error (RSE) [24]. *R*^2^ measures the proportion of variability in *Y* that can be explained using *X*. The adj-*R*^2^ is a modified version of *R*^2^ that has been adjusted for the number of parameters in the model. A fitted regression model uses the parameters to generate point estimate predictions. The differences between these predicted values and the ones used to fit the model are called ‘residuals’. The observed residuals are then used to subsequently estimate the variability in these values and to estimate the sampling distribution of the parameters. Theoretically, when the RSE is exactly 0, the model fits the data perfectly (likely due to overfitting). A better-performed model should obtain greater *R*^2^, especially greater adj-*R*^2^ and smaller RSE simultaneously. This article mainly introduces the application of four-parameter log-logistic model in the determination of biological activity *in vitro* and compares it with the fitting results of three-parameter log-logistic model. The advantages of four-parameter log-logistic model in the determination and analysis of bioactivity were illustrated.

## Materials and methods

### Cell culture

The mouse myoblast cell line C2C12 (ATCC, CRL-1772) cells were maintained in Dulbecco’s modified Eagle’s medium (HyClone, South Logan, UT, USA) supplemented with 10% fetal bovine serum (Gibco, Carlsbad, CA, USA), 1% Penicillin-Streptomycin Solution (Gibco), 2% 4-(2-Hydroxyethyl)-1-piperazineethanesulfonic acid (Gibco) at 37°C under 5% CO_2_. It is typical to passage cells every 1–2 days at a split ratio of 1:2 or 1:3, never allowing cells to reach >70% confluency. To examine the effects of rhBMP-2 in C2C12 cells, the growth medium was replaced with DMEM containing 2% FBS.

### ALP activity

Reconstitute the rhBMP-2 standard and rhBMP-2 sample with DMEM containing 2% FBS. This reconstitution produces 10 μg/ml of stock solutions for both standard and sample. When cells maintained in DMEM containing 10% FBS were at 70–80% confluency, aspirate the medium and rinse the cells by gently adding 1.5 ml of 0.25% trypsin-EDTA to T75 flask, so as not to dislodge cells. Remove the 0.25% trypsin-EDTA and incubate the flask for 2–3 min at 37°C, until the cells have begun to detach. Add 5 ml of warm DMEM containing 2% FBS to the flask and dissociate the cells by pipetting them up and down gently, and then transfer the cells to 50 wells of a 96-well plate at a density of 5000–10 000 cells per well. As for rhBMP-2 standard and sample, use the stock solution to produce a 2-fold dilution series of eight gradients. Mix each thoroughly before the next transfer. The undiluted stock solution (10 μg/ml) served as the highest concentration. Twenty-four hours after seeding, discard the medium and replace with DMEM containing 2% FBS and appropriate rhBMP-2 diluent in each well. Each rhBMP-2 concentration was assessed in triplicate. Seventy-two hours after incubation, aspirate each well and wash with 200 μl phosphate-buffered saline (PBS, HyClone Co.) for three times. After the last wash, remove any remaining PBS by aspirating or decanting. Invert the plate and blot it against clean paper towels. Add 100 μl cell lysis buffer (in 0.1 M glycine-NaOH buffer containing 1 mM MgCl_2_, 1 mM ZnCl_2_ and 1% Nonidet P40). Incubate for 1 h at room temperature. Add 50 μl of substrate solution (in 0.1 M glycine-NaOH buffer containing 4 mg/ml p-nitrophenyl phosphate). Incubate for 30 min at 37°C. Add 50 μl Stop Solution to each well. Determine the optical density of each well within 30 min, using a microplate reader set to 405 nm.

### Model fitting

The three-parameter log-logistic function is: $y=\frac{K}{1+ae^{-\mathrm{bx}}}$

Thus, three-parameter log-logistic regression fits three parameters, *K*, *a* and *b*, representing the maximum value of absorbance, (maximum-minimum)-1 and absorbance growth rate, respectively.

Specifically, the function of four-parameter log-logistic is: $y=D+\frac{A-D}{1+{\left(\frac{x}{C}\right)}^{-B}}$

This model includes four parameters, *D*, *A*, *C* and *B*. *D* and *A* indicate the minimum and maximum values of absorbance, respectively. *C*, generally been regarded as concentration for 50% of maximal effect (EC50), representing the concentration value at which the absorbance growth rate begins to change. *B* is the growth rate of absorbance, which is equivalent to the slope of the curve.

According to the least-square method, all parameters of both models were fitted by applying Gauss–Newton algorithm. The performance of fitted model were evaluated by *R*^2^, adj-*R*^2^ and RSE. A better performed model should obtain greater *R*^2^, greater adj-*R*^2^ and smaller RSE simultaneously. The model fitting was applied on RStudio, which can be downloaded from RStuio homepage (https://www.rstudio.com/products/rstudio/download/). The computing scripts are listed below:

# install and load “MASS” and “drc” packages

install.packages(“drc”)

library(MASS)

library(drc)

# prepare computing data from a csv file with experiments data

data<-read.csv(file.choose(), head=T)

# 3-parameter log-logistic model

mod.cl <- nls (Standard ∼ K/(1 + a*exp (-b*Concentration)), data = data, start = list (K = 3, a = 1, b = -1), trace = TRUE)

# 4-parameter log-logistic model

mod.ll4<- nls(Standard ∼ d+(a-d)/(1 + (Concentration/c)*(-b)), data = data, start = list (d = 0, a = 1, c = 1, b = -1), trace = TRUE)

## Results

### Using ALP activity as a quantified measurement indicator of rhBMP-2 bioactivity

In agreement to previous studies, ALP activity of C2C12 cells was significantly increased when exposed to rhBMP-2, and showed dose-dependent effects in a certain concentration range of rhBMP-2. The detection concentration range of our method was 0.078–10 μg/ml for rhBMP-2 standard substance. Then the actual bioactivity of samples can be calculated afterward based on utilized standard substance as well as fitted equations for both standard substance and sample. Considering the feasibility of quantifying ALP activity *in vitro*, it can be used as a measurement indicator of rhBMP-2 bioactivity.

### Logistic regression model fitting

As our data suggested, a sigmoid relationship between the ALP activity and rhBMP-2 concentration was revealed. To compare the effectiveness of different logistic models in coping such statistical problems, two models were selected and computed. All parameters of both models after fitting were listed below.

### Model goodness evaluation

To evaluate the overall performance of three-parameter log-logistic and four-parameter log-logistic models, *R*^2^, adj-*R*^2^ and RSE were taken into consideration. A better-performed model should obtain greater *R*^2^ and adj-*R*^2^ as well as smaller RSE simultaneously.

## Discussion

rhBMP-2 (dibotermin alfa) has been applied on surgically implantable medical devices, and results in the induction of new bone at the site of implantation when administered locally, which indicated rhBMP-2 as a potent treatment of bone defects. However, the role of BMP-2 on growth regulation also caused the concern about its carcinogenic potential. Thus, how to accurately quantify rhBMP-2 of rhBMP-2-loading medical devices both *in vitro* and *in vivo* became vital and fundamental. rhBMP-2 was firstly used as a biological product, and there are standardized quantification methods can be used for reference. Although considering the high risk and particularity of implantable devices, as well as the characteristics of varied rhBMP-2-loading carriers, the standard specification for rhBMP-2-loading medical devices requires further exploration and scrupulous validation. Here, we started with the aim to establish standard method for rhBMP-2 *in vitro* bioactivity quantification. Considering rhBMP-2 extracted from different methods usually varies in yield, purity and stability, it is not fully trustworthy to estimate the minimum effective amount of rhBMP-2 based on previous studies while most publications used protein level as the measurement of rhBMP-2 quantity. On the contrary, if the actual rhBMP-2 bioactivity was used for evaluating the effectiveness of rhBMP-2 in bone fracture treatment, it would provide a relatively unbiased platform for researchers to cross-validate with each other’s results. The greater adj-*R*^2^ and smaller RSE demonstrated that four-parameter log-logistic model beats three-parameter log-logistic model in fitting our data. Furthermore, with the adj-*R*^2^ >0.98, the accuracy of measurement is well-established. To sum up, by combining APL activity test and four-parameter log-logistic model fitting for both standard substance and samples, we can accurately quantify rhBMP-2 bioactivity *in vitro*, then the measured rhBMP-2 can be used for preparing rhBMP-2-loaded medical devices as well as making the comparable evaluation of the treatment. However, we have to admit that there is still a long way to go for completing the safety and effectiveness evaluation. For example, the toxicology studies including mutagenic potential, carcinogenic potential and immunogenicity shall be conducted.

**Table 1:** The results of different rhBMP-2 concentration and corresponding A405 values for both standard and sample

Concentration (10 μg/ml)	BMP-2 Standard	BMP-2 Sample
1	1.232 ± 0.005	1.177 ± 0.003
0.5	1.189 ± 0.014	1.148 ± 0.007
0.25	1.144 ± 0.016	1.131 ± 0.010
0.125	1.124 ± 0.003	1.049 ± 0.019
0.0625	1.019 ± 0.002	0.881 ± 0.084
0.03125	0.759 ± 0.051	0.504 ± 0.069
0.015625	0.431 ± 0.027	0.226 ± 0.056
0.007813	0.351 ± 0.041	0.181 ± 0.041

Data of triplicates are shown as mean ± SD.

**Table 2:** The results of parameter estimates in three-parameter log-logistic model

Parameters	Estimate	Standard error	*t*-value	*P*-value
*K*_std_	1.1691	0.0231	50.53	5.74e-08
*A*_std_	0.0231	0.0198	11.65	8.20e-05
*B*_std_	0.0178	0.0025	7.18	0.000818
*K*_sap_	1.1285	0.0234	48.21	7.25e-08
*A*_sap_	0.0379	0.0024	15.47	2.05e-05
*B*_sap_	0.0186	0.0022	8.58	0.000354

Parameters for rhBMP-2 standard and sample are indicated with subscripts ‘std’ and ‘sap’, respectively.

**Table 3:** The results of parameter estimates in four-parameter log-logistic model

Parameters	Estimate	Standard error	*t*-value	*P*-value
*D*_std_	−2.0973	0.4031	−5.20	0.006506
*A*_std_	0.2885	0.0566	5.10	0.006991
*C*_std_	1.1891	0.0222	53.45	7.344e-07
*B*_std_	0.0312	0.0029	10.63	0.000444
*D*_sap_	−2.2042	0.2161	−10.20	0.000521
*A*_sap_	0.1399	0.0278	5.04	0.007296
*C*_sap_	1.1550	0.0015	77.06	1.700e-07
*B*_sap_	0.0410	0.0019	21.04	3.016e-05

Parameters for rhBMP-2 standard and sample are indicated with subscript ‘std’ and ‘sap’, respectively.

**Table 4:** The results of model coefficients in both three-parameter log-logistic model and four-parameter log-logistic model

Coefficient	Three-parameter log-logistic		Four-parameter log-logistic	
	Standard	Sample	Standard	Sample
*R* ^2^	0.86837	0.91690	0.99550	0.99930
adj-*R*^2^	0.76965	0.85458	0.98950	0.99837
RSE	0.10978	0.10305	0.00375	0.00090

Data for BMP-2 standard and sample are indicated separately.

**Figure 1:**
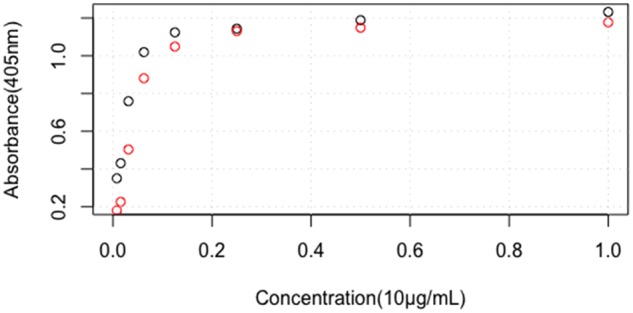
Dose-dependent effects of rhBMP-2 on ALP activity in C2C12 cells. The black circles indicate the rhBMP-2 standard product, and the red circles the rhBMP-2 sample. *X*-axis represents the graded concentrations of both rhBMP-2 sample and standard as described in Materials and methods section. *Y*-axis represents the absorbance of test wells at 405 nm. Data are presented as mean

**Figure 2:**
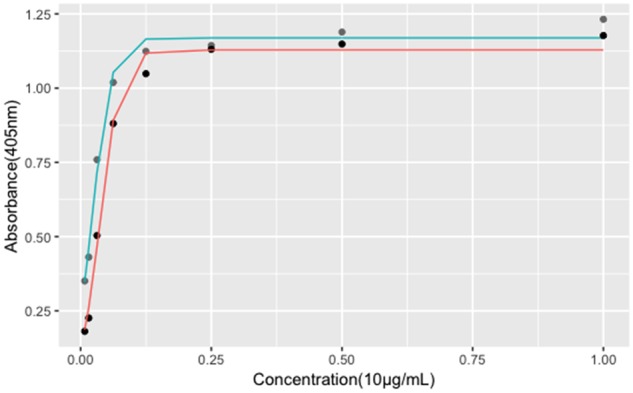
Fitting curves of three-parameter log-logistic model. Gray dots indicate the rhBMP-2 standard product, and black dots indicate the rhBMP-2 sample. The cyan curve represents fitting curve for standard under three-parameter log-logistic model, and the red curve represents for the sample. *X*-axis represents the graded concentrations of both rhBMP-2 sample and standard as described in Materials and methods section. *Y*-axis represents the absorbance of test wells at 405 nm. Data are presented as mean

**Figure 3:**
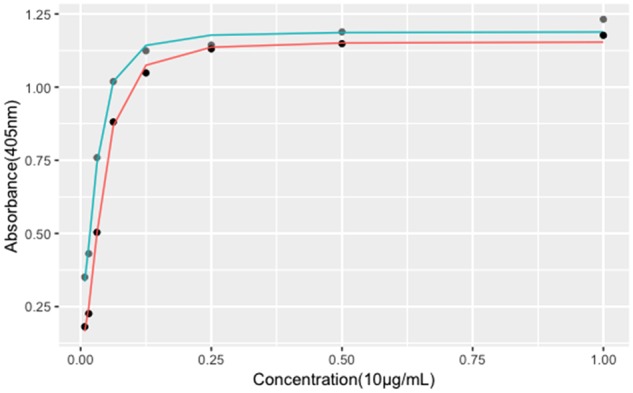
Fitting curves of four-parameter log-logistic model. Gray dots indicate the rhBMP-2 standard product, and black dots indicate the rhBMP-2 sample. The cyan curve represents fitting curve for standard under four-parameter log-logistic model, and the red curve represents for the sample. *X*-axis represents the graded concentrations of both rhBMP-2 sample and standard as described in Materials and methods section. *Y*-axis represents the absorbance of test wells at 405 nm. Data are presented as mean

## Conclusions

The four-parameter log-logistic model showed a superior performance of fitting compared to three-parameter log-logistic model with both greater *R*^2^, adj-*R*^2^ and smaller RSE. Thus, the four-parameter log-logistic model fits better than three-parameter log-logistic model for the sigmoid curves. In addition, the practical meaning of coefficients in four-parameter log-logistic model was easily interpreted. For instance, *D* and *A* indicate the minimum and maximum values of absorbance respectively. *C* represents the concentration value at which the absorbance growth rate begins to change. *B* is the growth rate of absorbance, which is equivalent to the slope of the curve. In order to make accurate estimations based on dose-response curve, dose points located in the region with the steepest slope were preferred. However, when considering the parallelity and assay-to-assay variability of a standardized quantification method, it may be preferable to use an assay design with doses distributed over a wide range and to apply a dose-response model which, like the four-parameter log-logistic, is capable of fitting over the whole feasible dose range. In this article, ALP activity test was proven to be a highly sensitive assay for *in vitro* rhBMP-2 bioactivity. Conversely, the potency of our statistic model also suggested the suitability of choosing ALP as rhBMP-2 testing indicator. Consequently, our results indicated rhBMP-2 quantification *in vitro* can be potently accomplished by detecting ALP activity and fitting four-parameter log-logistic model. This pipeline can serve as the groundwork of establishing serial rhBMP-2 standardized assays in medical devices. It is also worth noting that the combination of bioassay and statistical analysis is undoubtedly the trend of quantification methodology.

## Funding

This work was supported by the China National keypoint research and invention program of the thirteenth (Project ID: 2016YFC1102304).

*Conflict of interest statement*. None declared.
